# OrthoRehab: Development of a New Methodology for the Comparison Study Between Different Types of Ankle–Foot Orthoses in Foot Dysfunction

**DOI:** 10.3389/fdgth.2020.589521

**Published:** 2021-01-26

**Authors:** Cláudia Quaresma, Barbara Lopes, Jorge Jacinto, Tiago Robalo, Mariana Matos, Carla Quintão

**Affiliations:** ^1^Instrumentation, Biomedical Engineering and Radiation Physics Laboratory (LIBPhys-UNL), Physics Department, NOVA School of Science and Technology, NOVA University of Lisbon, Caparica, Portugal; ^2^Physics Department, NOVA School of Science and Technology, NOVA University of Lisbon, Caparica, Portugal; ^3^Rehabilitation Medicine Center of Alcoitão, Alcabideche, Portugal

**Keywords:** gait analysis, device, orthoses, rehabilitation, software, evaluation

## Abstract

Foot dysfunction is one of the most likely consequences of rheumatoid arthritis and stroke. It is characterized by severe changes in the gait pattern due to a significant increase in the plantar flexion. Some of these dysfunctions can be compensated by using an ankle–foot orthosis. However, the clinical decision about which orthosis best suits the patient creates a real problem for physicians/therapists.

**Purpose:** The main goal of this paper is to present a quantitative support tool that can assist the physicians/therapists in deciding which orthosis is most suitable for each subject.

**Methodology:** In order to achieve such goal, a platform named OrthoRehab was developed, and it was tested in three conditions: without any orthosis and with two different ankle–foot orthoses. The data were acquired in the Gait Laboratory of Rehabilitation Medicine Center of Alcoitão using a VICON NEXUS 1.8.5^®^ motion capture system that allows the capturing of kinematic and kinetic data.

**Results:** The results reveal that OrthoRehab is a user-friendly, easy to apply tool that analyzes very relevant data for the clinical staff.

**Conclusion:** The developed decision support tool, OrthoRehab, offers a quantitative analysis and provides insight to which orthosis achieves the best performance in comparison with the patient's gait pattern with no orthosis.

## Introduction

Major chronic diseases enumerated by the World Health Organization (WHO), for example, cancers, mental disorders, cardiovascular diseases, chronic respiratory diseases, and rheumatological diseases, have a huge impact on the quality of life of the individuals and represent the predominant health problems of the century (1, 2). Foot impairment is a major adverse condition in rheumatoid arthritis (RA) and stroke. Taking the total number of adult stroke patients into account, 10–20% have equinus foot as a severe consequence and >90% of patients with RA have reported foot complaints during the course of the disease such as hallux valgus (65%), longitudinal arch (42%) flattening, and claw toe (39%) (3–7). This is one of the most dysfunctional deformities with a significant impact on gait and quality of life of individuals who have suffered from stroke or RA (5, 6, 8–12). The possible treatment for these dysfunctions/deformities is the use of ankle–foot orthoses (AFOs) or, in severe cases, surgical intervention. AFOs are external biomechanical devices capable of improving the gait and physical functioning of the affected lower limb.

Traditionally, the AFOs are chosen based on the therapist's knowledge and clinical experience, patient's needs, or the qualitative analysis of the patient's gait (13). Moreover, the guidelines for AFO prescription provide a general recommendation and are not specific for each type of orthosis. For that reason, current clinical criteria for choosing a particular AFO are limited and subjective (14). This has a huge impact on a patient's gait performance while using the device (13).

Currently, there is a considerable research gap regarding the complete analysis of gait pattern, including the quantitative assessment of spatiotemporal, kinematic, and kinetic parameters, at the same time, for each patient during the use of AFO. Moreover, research is also missing about which orthosis best fits the functional needs of each subject (9, 15). Efficacy studies of AFOs to promote walking ability should be developed, and they will support physicians/therapists to make more precise and reliable decisions on the rehabilitation process (13). Therefore, this paper presents the OrthoRehab—a clinical decision tool that supports physicians/therapists to define AFO for a patient with foot dysfunctions during the rehabilitation process. Thus, the main goals of this paper are (1) to describe the development process of the OrthoRehab and (2) to present the results of its application in the clinical environment.

## Materials and Methods

OrthoRehab was designed and developed for a multidisciplinary team, composed of physicians, therapists, and biomedical engineers from Rehabilitation Medicine Center of Alcoitão (CMRA) and NOVA University of Lisbon. This study was approved by the Portuguese Ethics Committees of this Center.

Regarding the methodology used for the development of the OrthoRehab, the following steps were performed with the contribution of all team members:

1. Choice of the requirements of the platform
2. A decision on what gait parameters should be analyzed
3. Division of these parameters by categories: spatiotemporal, kinematic, and kinetic
4. Structuring the platform according to the categories defined in the previous step
5. Defining the calculation of the parameters under analysis
6. Programming the graphical interface.

In order to define the requirements, we asked potential end users, including the physicians and therapists who participated in this project, what facilities they thought would be important for the platform to have (16). The results of this consultation allowed identifying the following specific needs:

- easy to use: the tool should be quick to learn, and physicians/therapists should be able to use it
- should be in digital format
- facilitate the analysis of the parameters
- provide reports
- be compatible with VICON NEXUS gait analysis files.

A platform (OrthoRehab) was developed to meet these requirements. The graphical user interface was developed using MATLAB 2014b^®^ software. This allowed the introduction of VICON NEXUS 1.8.5^®^ (software used for gait acquisitions in this study) files and performing the analysis of the spatiotemporal, kinematic, and kinetic parameters.

Thus, the platform OrthoRehab was organized in the following sections:

1. Import/export files (file compatibility)
2. Calculation of parameters
3. Visualization of results
4. Report creation.

### Import/Export Files (File Compatibility)

The data acquisition is carried out using the software VICON NEXUS 1.8.5^®^ adapted to a computer. Since this new platform was designed to be compatible with this system, it does not increase any workload on clinicians other than importing the generated files into the developed graphical interface. After the introduction of the files in the software, the data are studied and analyzed. Finally, the outputs were saved in a database.

Although the VICON NEXUS 1.8.5^®^ software already analyzes all gait parameters, it considers only one gait cycle, in one condition (with or without orthoses), therefore being too general and unspecific.

### Calculated Parameters

The analyzed parameters, divided into spatiotemporal, kinematic, and kinetic (Table 1), were chosen in partnership with the team (physicians and therapists) from of the CMRA and according to the methodology used by Boudarham (7), Kinsella (17), and Manca (18).

**Table 1:** Analyzed spatiotemporal, kinematic, and kinetic parameters.

Spatiotemporal parameters	Step time and their asymmetry (s)	
	Step length and their asymmetry (m)	
	Step width and their asymmetry (m)	
	Velocity and global velocity (average velocity between both lower limbs) (m/s)	
	Cadence and their asymmetry (number of steps per minute)	
	Stance phase and their asymmetry (% gait cycle)	
	Swing phase (% gait cycle)	
	Single support phase and their asymmetry (% gait cycle)	
Kinematic parameters	Ankle joint's sagittal plane	Foot strike (”)
		Dorsiflexion's maximum (”)
		Plantar flexion's maximum (”)
	Ankle joint's coronal plane	Foot strike (”)
		Contralateral limb's foot off (”)
	Vertical extension between maximum and minimum of pelvis's sagittal plane (”)	
Kinetic parameters	Vertical ground reaction force	First maximum (N/kg)
		Second maximum (N/kg)
	Maximum of ankle joint's power (W/kg)	

First of all, each condition (with and without orthosis) is verified whether the gait tests have adequate dynamic data of both lower limbs, that is, if there is at least a gait cycle without artifacts on the force platforms. Then, all the parameters listed below (Table 1) are calculated for each condition and for each gait cycle, and the average value of the parameters is determined for each condition. Finally, all values and curves are shown in the graphical interface corresponding to the average of all running cycles for the lower limb under analysis.

#### Spatiotemporal Analysis

The spatiotemporal parameters analyzed in each gait cycle are those related to time intervals, distances, velocity, cadence, and respective asymmetries. These asymmetries correspond to the differences in the values of each lower limb.

For all conditions under analysis, each spatiotemporal parameter is calculated, and its values are firstly compared with normative ones. Afterward, the condition in which the patients show the best performance (i.e., higher velocities and lower asymmetries, is highlighted).

#### Kinematic Analysis

The kinematic analysis curves correspond to the average value over the gait cycles of the angles of movement by the percentage of each cycle.

The kinematic parameters analyzed by the OrthoRehab are:

- Sagittal plane of the tibiotarsal joint—defined by the points: initial contact, maximum dorsiflexion, and maximum plantar flexion
- Frontal plane of the tibiotarsal joint—defined by the points: initial contact and the release of the fingers of the contralateral lower limb
- Sagittal plane of the pelvis—defined by the vertical extension between the maximum and minimum points.

For ankle joint and ground reaction force, the developed interface shows charts with angle and force curves, respectively, for at most three conditions (without orthosis and with two different types of orthoses, generically denominated A and B). The kinematic analysis identifies which condition (without orthosis, with orthosis A or orthosis B) allows an increase in dorsiflexion amplitude, a decrease in the varus, and a lower energy expenditure of the gait of the individuals under analysis.

#### Kinetic Analysis

The kinetic parameters analyzed by the program are those related to force and energy, namely, vertical ground reaction force and a maximum of ankle joint's power.

The dynamic parameters analyzed by the program are:

- The vertical reaction force of the soil—the analyzed points are the first and second maxima
- Strength of the tibiotarsal joint—the point analyzed is the maximum of the curve that occurs just before the foot leaves the ground (of the lower limb under analysis). The purpose of studying the vertical force of the ground reaction is to assess the individual's ability to exert force on himself.

The dynamic analysis curves correspond to the mean value of the force per percentage of support phase and power and per cycle percentage. The vertical extension of the pelvis and the ankle joint force values are calculated for the three conditions and the lowest and highest values, respectively, are selected and highlighted. The values are presented in tables.

### Graphical User Interface Visualization of Results

Since the visualization of the results is an essential issue for the usability of the interface, the graphical interface architecture and the functionalities were defined with physicians and therapists using a co-creation methodology.

#### Interface Design Requirements

The graphical interface was developed to fulfill the following requirements:

- Utility and functionality—should perform the calculations on the parameters of interest and functionally presents them
- Sequential and intuitional—all relevant information should be distributed in a simple, sequential, and intuitive way
- Innovative—an original tool that allows a comparative analysis between clinical results obtained for different types of orthoses
- Without additional work for physicians and therapists.

#### Graphical Interface Architecture

The graphical interface developed has an architecture that provides the following main sections: Main Menu, Import Files, and three tabs for data analysis: Space–Time Analysis; Kinematic Analysis; Dynamic Analysis.

**Figure 1:**
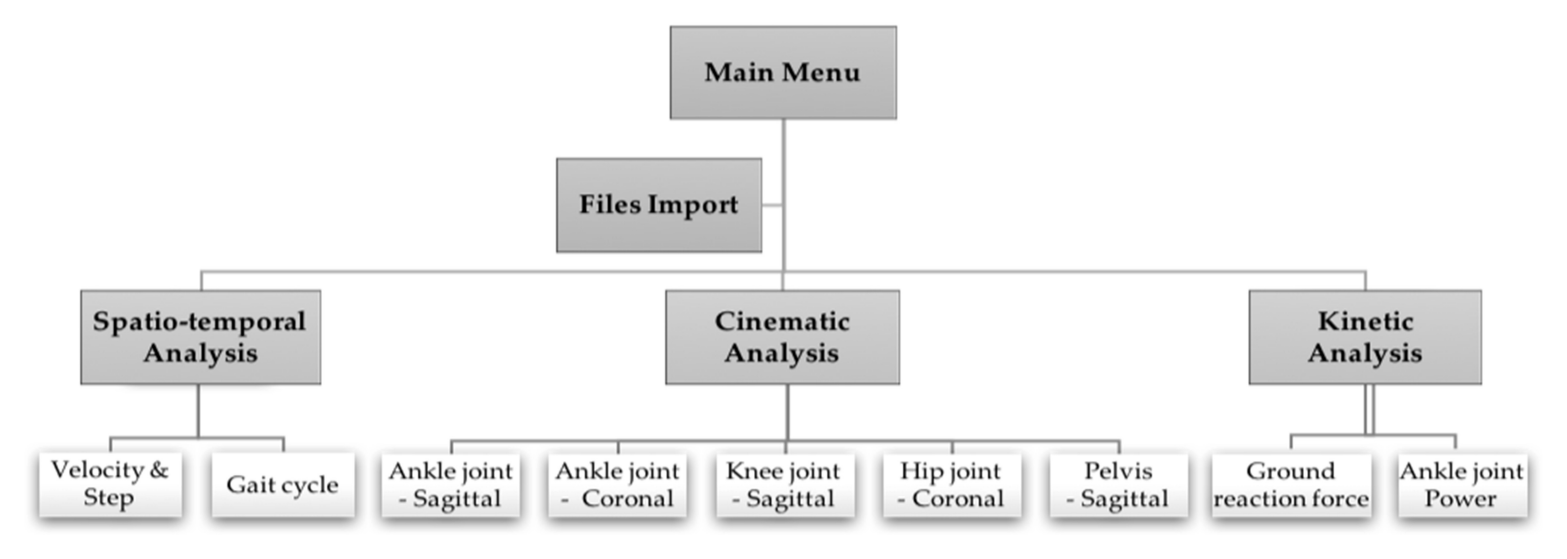
Program structure.

The parameters under analysis are shown in tables and graphs. The program starts when the Main Menu window (Figure 1) opens. This window allows the user to introduce the data of each patient and import the files to be analyzed. After the files are imported, it is possible to access the three available types of analysis. Also, a support manual was also prepared to understand the design of the program, as well as all its functionalities.

#### Functionalities

The graphic interface developed has the following functionalities:

- Ability to read files in CSV format produced by VICON NEXUS 1.8.5^®^ software
- Perform spatiotemporal, kinematic, and kinetic analyses in three possible conditions: without orthosis, with orthosis A and with orthosis B—although the program is idealized to analyze three conditions, it is possible to perform the analyses with only two conditions under study
- Possibility to analyze one or both lower limbs
- Show the mean value of the parameters under analysis
- Present in the form of a table or graph the parameters analyzed for both lower limbs in the different conditions
- Highlight values that are closer to the normative standard (for example, higher speed) whenever possible
- Selection of the name of the dynamic orthoses under study, with no restriction for the type of the orthoses
- The option of activation of the non-pathological pattern in the graphs
- Graphical interactivity—zoom in and zoom out of the graphs, as well as display coordinates of points of the curves by placing the cursor
- Data recording capability
- Creation of a database (XLSX format) organized with all the parameters of interest
- Automatic addition of data from individuals to the database.

## Pilot Study

The OrthoRehab tool was tested in three participants (two females and one male) with equinus foot dysfunction caused by a stroke (left hemiplegia). None of the subjects had previous experience with any of the orthoses under test (Table 2).

**Table 2:** Participant's data.

**Subject**	**1**	**2**	**3**
Gender	Male	Female	Female
Age (years)	47	40	67
Height (±0.05 cm)	179.50	163.00	149.00
Body mass (±0.1 kg)	70.4	67.0	65.0
Time since stroke (days)	95	420	62
Ankle joint tone (1–4)	2	1	1
Passive joint range (Modified Ashword Scale)	Ankle joint dorsiflexion: +- 0”	No limitations	Ankle joint dorsiflexion: +- 0”
FAC - functional ambulatory category (1–6)	1	3	3
Dynamic balance in standing position (0–56) (Berg Balance Scale)	15	48	45

The inclusion criteria are the diagnosis of stroke with injury only in the right hemisphere (ability to understand and speech not compromised); equine foot, with dorsiflexion of the tibiotarsal joint up to 0° passively and modified Ashworth scale with scores: 0, 1, 1+, or 2; age between 55 and 65 years (excluding young strokes and degenerative motor disorders characteristic of older ages); ability to carry out independent walking by third parties in the minimum distance of 10 m, being allowed the use of a walking aid; ability to walk with shoes but without orthosis; the initial phase of training with orthosis: five training sessions or <1 week of use.

### Procedure

Each subject was previously informed about the procedures and the objectives of the study and signed an informed consent.

The gait acquisitions were performed in the Biomechanics Laboratory of CMRA with controlled conditions of temperature and light and a regular floor. The acquisition procedure was performed in four steps:

1. Measurement of anthropometric parameters such as weight, height, distance between iliac crests, leg length, knee width, and ankle width
2. The software requires the placement of 16 reflective markers (eight on each lower limb) in specific anatomical places according to standard protocols for a lower body motion analysis using the Vicon Plug-In Gait Model. In total, 16 reflective markers are placed, eight on each lower limb. These are placed in medial and lateral locations of the joints that are considered anatomical landmarks (19, 20).
3. The participants, wearing shoes, walked along the force platforms at a speed that they considered comfortable in the three conditions: (I) with no orthosis; (II) with orthosis A (posterior support); (III) with orthosis B (anterior support).
4. Point 3 was repeated two more times for each condition.

Kinetic data were collected with four force platforms from Advanced Mechanical Technology, Inc., AMTI OR6-7-2000 (50.8 × 46.4 cm) with four analogical amplifiers AMTI that were longitudinally oriented and embedded flush with the ground. Kinematic data were collected by six infrared cameras VICON T-Series T10 (1 megapixel). Additionally, there were two digital video cameras Basler piA1000-48gc GigE. All the equipment was connected to VICON NEXUS 1.8.5^®^ software that allowed the simultaneous collection of kinematic and kinetic data of the gait of the subjects under analysis at a frequency of 100 Hz for infrared cameras and 1,000 Hz for force platforms.

## Results

In order to demonstrate the feasibility of OrthoRehab, as mentioned above, it was applied to three patients by a physician and a therapist. In this section, we present the end users' comments and the results from the pilot study.

### End Users' Comments

The physician and the therapist who applied the OrthoRehab tool, during the proof-of-concept process, indicated that the tool is easy to be applied and it can support them to choose the most suitable type of orthosis according to the patient's needs. Additionally, the users mentioned that OrthoRehab can be used without workload and provide an evaluation that they cannot have in a conventional approach. Moreover, a high level of consensus was found with regard to whether there was a clinical need for the proposed new tool.

There was, also, a high level of agreement that the key to the success of the OrthoRehab was including physicians and therapists in the co-creation of this tool.

### OrthoRehab Application

The analysis was performed only in the gait cycles of the left lower limb (affected limb) in the three conditions using OrthoRehab tool.

The average values, taking into account the three participants and the three trials performed for each one of them, were calculated.

### Spatiotemporal Parameters

For velocity analysis, the highest value was selected. All participants presented values that were much lower than those referred to as non-pathological: 1.3 m/s (19). However, on average, the highest velocity was observed when the patients were using orthosis B.

Regarding the asymmetry of the cadence, the results show that A is the orthosis that causes the lowest values, while orthosis B reduced the asymmetry of time and step width. For step length, none of the orthoses proved to be efficient.

To reduce stance phase asymmetry, B is the orthosis that shows more benefits. To decrease the single support phase asymmetry, the best choice is A. In all participants, an increase of stance phase and a decrease of single support phase were also verified for both orthoses.

### Kinematic Parameters

Maximum dorsiflexion and maximum plantar flexion of the sagittal plane of the ankle joint are taken into account to evaluate the increase of dorsiflexion. The gait performance was considered improved when the values of interest taken from the curves with orthosis are higher when compared with the values taken from the curves without orthosis.

**Figure 2:**
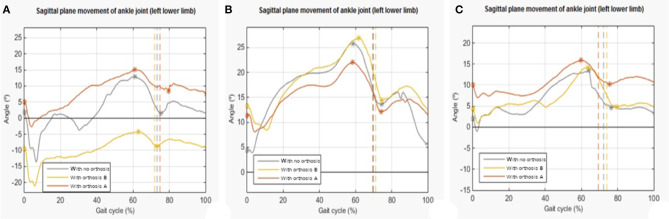
Examples of plots representing the sagittal plane movement of the ankle joint of the pathological lower limb in the three conditions in the analysis. **(A)** Trial 1 of subject 1. **(B)** Trial 1 of subject 2. **(C)** Trial 1 of subject 3. The vertical line presents the contact phase, and the * is the higher value.

The angular results show that, on average, A is the orthosis that increases dorsiflexion [an increase of 6.133° (±0.001°) in foot impact, 0.614° (±0.001°) in maximum dorsiflexion, and 3.805° (±0.001°) in maximum plantar flexion]. Only subject 2 shows different results (Figure 2).

The increase in the stance phase was evaluated in order to analyze temporal behavior. The results obtained show that, on average, the use of orthoses does not improve temporal variation.

For angular analysis of foot impact and contralateral raised foot in the coronal plane of the ankle joint, we evaluated the increase in the *varus*.

**Figure 3:**
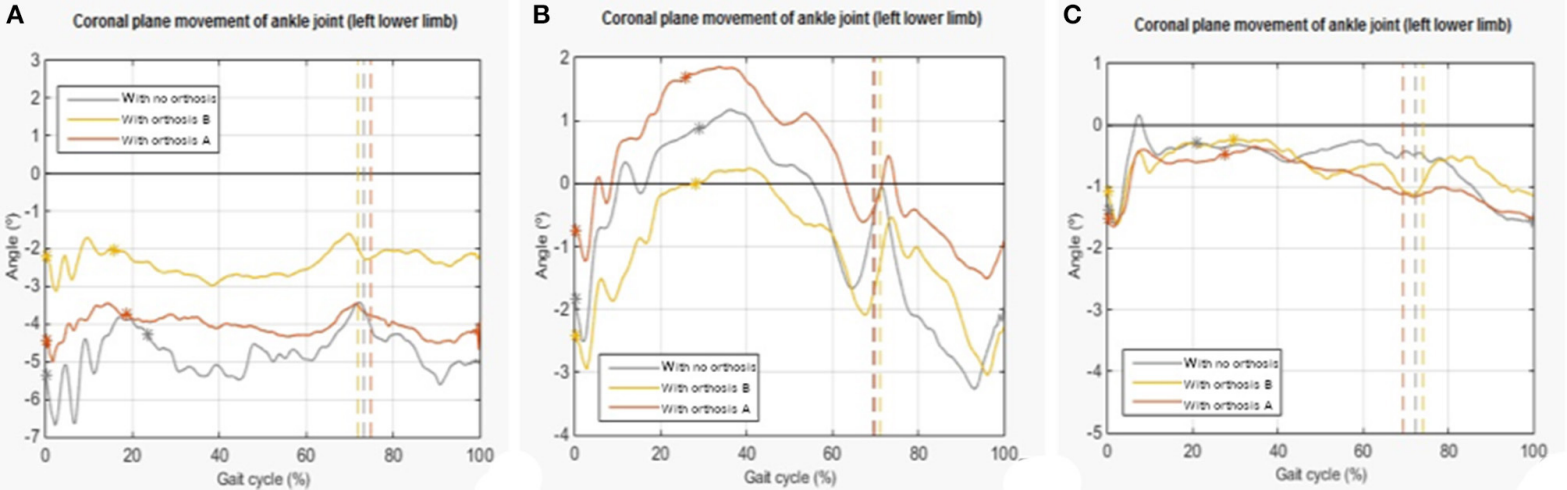
Examples of plots representing the ankle joint's coronal plane of the pathological lower limb in the three conditions in the analysis. **(A)** Trial 1 of subject 1. **(B)** Trial 1 of subject 2. **(C)** Trial 1 of subject 3. The vertical line presents the contact phase, and the * is the higher value.

The angular results show that, on average, both orthoses can decrease the characteristic *varus* of equinus foot dysfunction. However, the orthosis that shows quantitatively more significant improvement is B [an increase of 0.768° (±0.001°) in foot impact and 0.394° (±0.001°) on raised foot of contralateral limb]. Once again, subject 2 shows different results (Figure 3).

About the temporal variation, the methodology used is the same as the one used for the sagittal plane. Once again, the results show that, on average, the use of orthosis does not improve temporal variation.

The vertical extension of the angular pelvis in the sagittal plane is directly related to the gait's energy expenditure. We look for the lowest values of extension, since they mean lower energy expenditure and consequently more efficient gait. Although the data show that both orthoses impart a beneficial effect, on average, the A orthosis produces better results.

### Kinetic Parameters

Concerning the ground reaction force, the higher force produced better performance. When the first and the last peaks were analyzed, on average, only A shows positive results in both peaks.

Regarding temporal variation, we can say that there is an improvement in the first peak of the curve if it is on the left of the corresponding peak without orthosis, and there is an improvement in the second peak if it is on the right. This criterion is adopted because it is verified, in the analyzed cases, that the first peak occurs later and the second peak occurs earlier relative to non-pathological behavior. The results show that, on average, no orthosis improves the first peak temporally and A shows benefits in the second peak (Table 3). It is also observed that the temporal relationship between the first and the second peak is much lower than the standard value of reference: 60% (21).

**Table 3:** Mean values of the affected lower limb at the moment when the two peaks of reaction force of the soil occur.

		**Average value (±0.01% support phase)**		**Normative reference value [17]**
		**Subject 1**	**Subject 2**	
1st Peak	With no orthosis	48.01	42.37	20%
	Orthosis B	50.28	47.47	
	Orthosis A	49.79	50.25	
2nd Peak	With no orthosis	64.74	58.25	90%
	Orthosis B	67.59	56.00	
	Orthosis A	66.64	58.00	
Difference between the 1st and 2nd peak	With no orthosis	16.73	15.88	60%
	Orthosis B	17.31	8.52	
	Orthosis A	16.85	7.75	

For the maximum force of the ankle joint, which occurs immediately before the raised foot, the results show that, on average, none of the orthoses can improve this parameter.

## Discussion/Conclusions

In this paper, we present the development of a decision support tool called OrthoRehab, as well as the steps involved in this process. Performance of the tool was evaluated in a clinical environment. Regarding the usability of the tool, the clinical team considered that OrthoRehab was easier to use without workload as well as user-friendly.

OrthoRehab provides simultaneous analysis for each subject in three conditions: with no orthosis and with two different AFOs. This tool gives a comparative and quantitative analysis of the most relevant gait parameters for foot dysfunction. Therefore, OrthoRehab contributes to the development of clinical plans for each subject with this dysfunction and, more specifically, it helps with the correct dynamic AFOs prescription.

The tool development methodology began with the decision about what were the most valuable kinematic and kinetic parameters for analysis of foot dysfunction and their distribution categories. This allowed developing a software that analyzes these parameters and plots them using an appropriate graphical interface. These two items (parameters and plots) provide physicians and therapists a global, integrated, and innovative evaluation of patient's gait in relation to the orthoses under study.

OrthoRehab was applied in a real clinical context, and it proved to be a reliable and suitable tool, fulfilling the objectives that were established.

OrthoRehab will help physicians and therapists in making a better, personalized, and reasoned decision about the AFO prescription. This tool can be useful in different clinical areas such as rheumatology, ortho-traumatologic, and also in populations of varying ages, since it can be applied to any person who has been prescribed a lower limb orthosis. Additionally, the OrthoRehab can be adapted to be applied to other dysfunctions/deformities and/or with different orthoses.

## Data Availability Statement

The raw data supporting the conclusions of this article will be made available by the authors, without undue reservation.

## Ethics Statement

The studies involving human participants were reviewed and approved by Rehabilitation Medicine Center of Alcoitão. The patients/participants provided their written informed consent to participate in this study.

## Author Contributions

CQua, BL, JJ, and CQui contributed to the conception, design of the study, and participated in drafting the final manuscript. BL performed the tool and analyzed the data. All authors contributed to the conception, design of the review, contributed to manuscript revision, read, and approved the submitted.

## Conflict of Interest

The authors declare that the research was conducted in the absence of any commercial or financial relationships that could be construed as a potential conflict of interest.
